# Evaluating nursing faculty's approach to information literacy instruction: a multi-institutional study

**DOI:** 10.5195/jmla.2020.841

**Published:** 2020-07-01

**Authors:** Bethany S. McGowan, Laureen P. Cantwell, Jamie L. Conklin, Rebecca Raszewski, Julie Planchon Wolf, Maribeth Slebodnik, Sandra McCarthy, Shannon Johnson

**Affiliations:** 1 bmcgowa@purdue.edu, Library of Engineering and Science, Purdue University in West Lafayette, IN; 2 lcantwell@coloradomesa.edu, Tomlinson Library, Colorado Mesa University, Grand Junction, CO; 3 jconklin@unc.edu, Health Sciences Library, University of North Carolina at Chapel Hill, Chapel Hill, NC; 4 raszewr1@uic.edu, Library of the Health Sciences, University of Illinois at Chicago, Chicago, IL; 5 jspw@uw.edu, Bothell Campus Library, University of Washington Bothell and Cascadia College, Bothell, WA; 6 slebodnik@email.arizona.edu, Health Sciences Library, University of Arizona, Tucson, AZ; 7 mccarthy@wccnet.edu, Bailey Library, Washtenaw Community College, Ann Arbor, MI; 8 johnsons@pfw.edu, Library, Purdue University Fort Wayne, Fort Wayne, IN

## Abstract

**Objective:**

In 2018, the Association of College & Research Libraries (ACRL) Health Sciences Interest Group convened a working group to update the 2013 Information Literacy Competency Standards for Nursing to be a companion document to the 2016 Framework for Information Literacy in Higher Education. To create this companion document, the working group first needed to understand how nursing faculty approached information literacy (IL) instruction.

**Methods:**

The working group designed a survey that assessed how nursing faculty utilized IL principles in coursework and instruction. The survey consisted of nineteen mixed methods questions and was distributed to nursing faculty at eight institutions across the United States.

**Results:**

Most (79%) faculty indicated that they use a variety of methods to teach IL principles in their courses. While only 12% of faculty incorporated a version of the ACRL IL competencies in course design, they were much more likely to integrate nursing educational association standards. Faculty perceptions of the relevance of IL skills increased as the education level being taught increased.

**Conclusion:**

The integration of IL instruction into nursing education has mostly been achieved through using standards from nursing educational associations. Understanding these standards and understanding how faculty perceptions of the relevance of IL skills change with educational levels will guide the development of a companion document that librarians can use to collaborate with nurse educators to integrate IL instruction throughout nursing curriculums at course and program levels.

## INTRODUCTION

### Background

Since their introduction in 2013, the Association of College & Research Libraries (ACRL) Information Literacy Competency Standards for Nursing have served as an authoritative resource for librarians who are developing nursing instruction [1]. These standards were developed by reviewing standards from nursing educational associations for baccalaureate, master's, and doctoral programs [2]. The five standards include performance indicators and outcomes for demonstrating nurses' information literacy (IL).

In January 2016, the ACRL adopted the Framework for Information Literacy for Higher Education that, rather than relying on standards or learning outcomes, focused on core concepts that provide flexibility for implementation [3]. Due to the development of the Framework, the ACRL Board of Directors voted to rescind the Information Literacy Competency Standards for Higher Education, meaning that the 2013 Information Literacy Competency Standards for Nursing must now be revised to reflect the new framework structure.

In January 2018, the ACRL Health Sciences Interest Group (HSIG) convened a working group to revise the Information Literacy Competency Standards for Nursing. This working group included nursing liaison librarians from various academic institutions, along with an ACRL liaison representative. To understand how nursing faculty approached IL instruction, the group began with a preliminary review of related literature. The findings from this review, combined with the groups' expertise as nursing liaison librarians, were used to design and execute a mixed-methods survey that examined nursing faculty's familiarity with IL principles and standards, perceptions of IL relevance to nursing education, and perceptions of IL relevance based on the educational level being taught.

We wanted to understand how nursing faculty integrated IL instruction into nursing educational curriculums and their familiarity with the ACRL IL Framework or Standards. If they were familiar with either the ACRL Framework or Standards, we wanted to know how these were being used in course curriculum design. We hypothesized that nursing faculty utilized nursing standards and the ACRL Information Literacy Competency Standards for Nursing in curriculum development for IL instruction but were less familiar with the newer, broader ACRL Framework. The underlying assumption for this hypothesis was that nursing faculty would be more familiar with established nursing standards. This research project presents survey findings that could be applicable for librarians who are collaborating with nursing faculty to teach IL skills.

### Nursing standards supporting information literacy (IL) instruction in nursing education

Several nursing educational associations provide standards that support IL instruction. Familiarity with these nursing-centric perspectives can help librarians develop the language and arguments needed to discuss the arc of IL-related concepts and skills in nursing education curriculums.

#### The American Association of Colleges of Nursing Essentials.

Nursing education accrediting agencies, such as the Commission on Collegiate Nursing Education and the accrediting body of the American Association of Colleges of Nursing (AACN), have identified nine baccalaureate essentials to prepare nursing graduates for a future in health care. These are commonly referred to as the “Essentials.” The Essentials guide nurse educators in the “necessary curriculum content and expected competencies of graduates from the baccalaureate, master's, and doctor of nursing practice programs, as well as the clinical support needed for the full spectrum of academic nursing” [4].

The current baccalaureate Essentials were adopted in 2008 and are in the process of being revised by a task force of nursing professionals, with a draft document released in May 2020 [5]. Emphasis on the ability to execute IL-related competencies plays a role throughout all nine of the Essentials, but IL is specifically mentioned in “Essential I. Liberal Education for Baccalaureate Generalist Nursing Practice: A solid base in liberal education provides the cornerstone for the practice and education of nurses”; “Essential III: Scholarship for Evidence-Based Practice: Professional nursing practice is grounded in the translation of current evidence into one's practice”; “Essential IV. Information Management and Application of Patient Care Technology: Knowledge and skills in information management and patient care technology are critical in the delivery of quality patient care”; and “Essential IX. Baccalaureate Generalist Nursing Practice: The baccalaureate graduate nurse is prepared to practice with patients, including individuals, families, groups, communities, and populations across the lifespan and across the continuum of healthcare environments. The baccalaureate graduate understands and respects the variations of care, the increased complexity, and the increased use of healthcare resources inherent in caring for patients.” The baccalaureate graduate understands and respects the variations of care, the increased complexity, and the increased use of health care resources inherent in caring for patients [6].

#### The Quality and Safety Education for Nurses Project.

The Quality and Safety Education for Nurses (QSEN) project is managed by the AACN QSEN Education Consortium, with funding from the Robert Woods Johnson Foundation [7]. The project aims to prepare future nurses to support safe, high-quality health care systems. The six QSEN competencies are based on recommendations from the Institute of Medicine Health Professions Education report [8], adjusted for use in nursing pre-licensure programs. QSEN competency guides are available for nursing education at the undergraduate level, which were released in 2007 [9], and at the graduate level, which were released in 2009 and updated in 2012 [10].

Both guides include definitions for each competency along with the required knowledge, skills, and attitudes. IL concepts are clearly reflected in “Competency III. Evidence-Based Practice: Integrate best current evidence with clinical expertise and patient/family preferences and values for delivery of optimal health care”; “Competency IV. Quality Improvement: Use data to monitor the outcomes of care processes and use improvement methods to design and test changes to continuously improve the quality and safety of healthcare systems”; and “Competency VI. Informatics: Use information and technology to communicate, manage knowledge, mitigate error and support decision making.”

#### The Technology Informatics Guiding Educational Reform Competencies Model.

The Technology Informatics Guiding Educational Reform (TIGER) Competencies Model [11], released in 2009 by the TIGER Informatics Competencies Collaborative, consists of competencies related to computer literacy, IL, and information management, with the IL component based on the ACRL Information Literacy Competency Standards. The competency recommendations are that practicing nurses and graduating nursing students are able to:

- determine the nature and extent of the information needed;
- access needed information effectively and efficiently;
- evaluate information and its sources critically and incorporate select information into his or her knowledge base and value system;
- individually or as a group member, use information effectively to accomplish a purpose; and
- evaluate outcomes of the use of information.

These nursing standards provide insight into how nurse educators understand IL and approach IL instruction. Familiarity with these standards offers librarians the opportunity to converse more knowledgeably and leverage collaborations with nursing faculty.

## METHODS

### Survey instrument and overview

The ACRL/HSIG Nursing Information Literacy Framework Working Group used Melnyk's seven evidence-based practice steps [12], literature review findings, and their combined experiences as nursing liaison librarians to design a survey that assessed how nursing faculty utilized IL concepts in coursework and instruction. This included the development of seven IL skills comprising a mixture of lower-level and higher-level cognitive skills. Faculty were asked to rate the relevance of each skill based on level of education.

We believed that distributing the survey internally would result in higher response rates than distributing to an external audience would and that the combined faculty from our eight institutions provided a representative sample. Each member of the working group distributed the survey to nursing faculty at their institutions. The survey was distributed via email, and responses were collected and stored using Qualtrics survey software. The survey consisted of nineteen mixed methods questions and included questions about respondents' educational level, curriculum involvement, familiarity with IL standards, incorporation of IL into teaching practices, and perspectives on the relevance of IL across education levels. It was open for responses from November 1, 2018, to January 7, 2019. The survey instrument is provided in supplemental Appendix A.

Institutional review board (IRB) exemptions or approvals were granted for each of the institutions in which nursing faculty were surveyed. The IRB approvals are from Colorado Mesa University, #19-22; Frontier Nursing University; Purdue University (West Lafayette and Fort Wayne), #1809021023; University of Illinois at Chicago #2018-1389; University of North Carolina at Chapel Hill; and University of Washington, #STUDY00005931. North Central Michigan College allowed surveying of faculty without IRB approval.

### Participants

To capture diverse and inclusive perspectives, the Nursing Information Literacy Framework Working Group was crafted to represent a mixture of types of higher education institutions, including community colleges, teaching colleges, and research universities. These institutions also represented a mix of US regions including the Pacific Northwest, Pacific Southwest, Midcontinental, Greater Midwest, and Southeast/Atlantic regions. Members of the working group were responsible for recruiting nursing faculty at their respective institutions and utilized departmental email discussion lists and departmental faculty meetings to recruit faculty to participate in the survey.

A total of 512 nursing faculty at 8 institutions received recruitment emails from the working group member who served as their nursing liaison librarian. These institutions included Colorado Mesa University, Frontier Nursing University, North Central Michigan College, Purdue University, Purdue University Fort Wayne, University of Illinois at Chicago, University of North Carolina at Chapel Hill, and University of Washington. The recruitment email included an anonymized link to the survey. A sample recruitment email is available in supplemental Appendix B.

### Data analysis

Survey responses were exported from Qualtrics to Excel, where they were cleaned and a codebook was created. The codebook is provided in the Purdue e-Pubs data repository as noted in the Data Availability Statement. Excel was used to visualize survey results.

## RESULTS

Eighty-seven faculty participated in the survey, and of these, 68 completed the entire survey. After incomplete survey submissions were removed, our response rate was 13%. The highest degree held by the majority of respondents was a doctorate (PhD) in nursing (n=29), and 14 held a PhD in another discipline. Ten faculty held a doctor of nursing (DNP) degree, and 15 held a master's degree in nursing (MSN). A tabular summary of survey responses, including participant demographic details, is available in supplemental Appendix C.

Most faculty (87%, n=59) indicated that their institutions had curriculum committees in their nursing programs that generated student learning objectives and course outcomes. However, only 51% (n=35) indicated that their nursing programs had a goal related to IL. An additional 37% (n=25) were not sure if their programs had an IL goal.

Most faculty (69%, n=47) have been involved with their nursing programs' curriculum. Some faculty reported multiple roles in their curriculum involvement. Some (26%, n=18) currently served as curriculum committee members, 26% (n=18) had served as curriculum committee members in the past, and 15% (n=10) had served in a leadership role on curriculum committees. In addition, 13% (n=9) had served on their programs' accreditation committees, 12% (n=8) had served on assessment committees, and 15% (n=10) had served in another similar capacity.

### Faculty perceptions of IL relevance, based on educational level

Of the 68 faculty respondents, 56% (n=38) taught at the baccalaureate level, followed by 37% (n=25) at the DNP level, 34% (n=23) at the MSN level, 12% (n=8) at the PhD level, and 1% (n=1) at the associate level; 3% (n=2) were not currently teaching. Nursing faculty were asked to use a 5-point scale (1=not at all relevant, 5=extremely relevant) to rate the relevance of 7 IL skills at the associate, baccalaureate, master's, DNP, and PhD educational levels.

**Figure 1:**
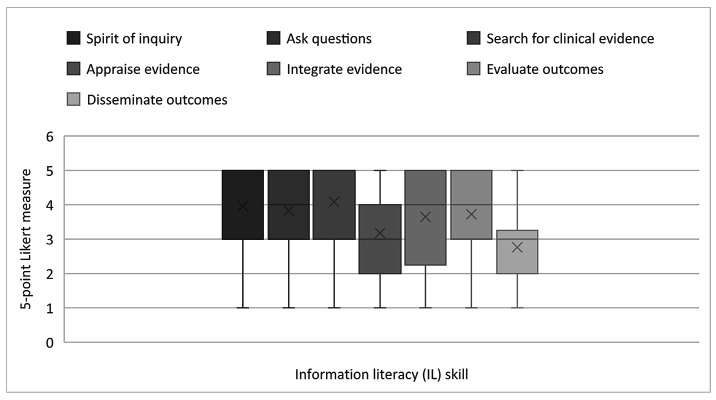
Nursing faculty ratings of the relevance of information literacy (IL) skills at the associate level

At the associate level, faculty rated the ability to cultivate a spirit of inquiry, ask burning clinical questions, search for the best and most relevant clinical questions, and evaluate outcomes between 3 (moderately relevant) and 5 (extremely relevant) on a 5-point scale. This 2-point difference in response averages suggested significant variation in faculty opinions. Regarding the relevance of student ability to integrate evidence with clinical expertise, there was even more variation in faculty opinions, with a nearly 3-point difference in response averages, ranging between ~2 (slightly relevant) and 5 (extremely relevant). Higher level cognitive skills were viewed as less relevant for associate-level students, including the ability to critically appraise evidence (average responses between 2 [slightly relevant] and 4 [very relevant]) and the ability to disseminate outcomes (average responses between 2 [slightly relevant] and 3 [moderately relevant]. Compared with the other educational levels in the survey, the associate level was associated with the most variation in faculty perceptions of the relevance of IL skills. Figure 1 illustrates nursing faculty perceptions of the relevance of IL skills at the associate level.

**Figure 2:**
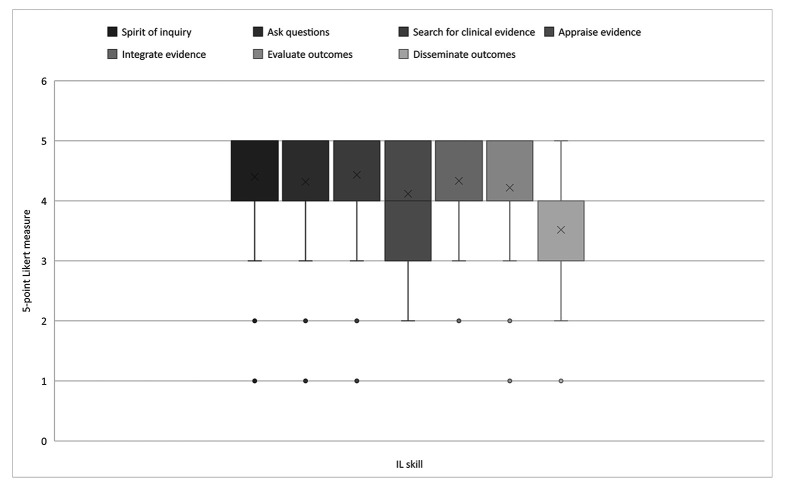
Nursing faculty ratings of the relevance of IL skills at the baccalaureate level

At the baccalaureate level, faculty rated 5 of the 7 IL skills as highly relevant, with average scores between 4 (very relevant) and 5 (extremely relevant). These skills included the ability to cultivate a spirit of inquiry, ask burning clinical questions, search for clinical evidence, integrate evidence, and evaluate outcomes. Faculty also perceived the ability to appraise evidence as relevant, but their responses varied, with average scores ranging between 3 (moderately relevant) and 5 (very relevant). The ability to disseminate outcomes was seen as the least relevant at this educational level, with scores ranging between 3 (moderately relevant) and ~4 (very relevant). These results illustrated a shift in expectations with the increased educational level, as nursing faculty were more in agreement with each other and rated IL skills as more relevant at the baccalaureate level than at the associate level. Figure 2 illustrates nursing faculty perceptions of the relevance of IL skills at the baccalaureate level.

**Figure 3:**
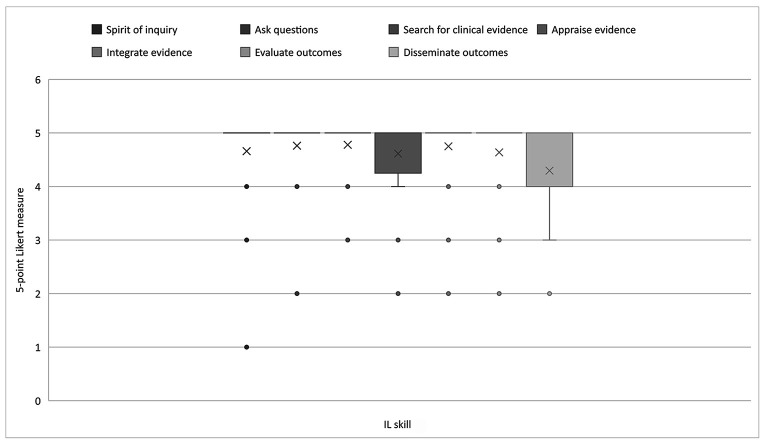
Nursing faculty ratings of the relevance of IL skills at the master's level

At the master's level, there was an even more pronounced shift in faculty perceptions of IL relevance, with nursing faculty mostly agreeing that all 7 IL skills were very or extremely relevant. Faculty nearly unanimously rated the ability to cultivate a spirit of inquiry, ask questions, search for clinical evidence, integrate evidence, and evaluate outcomes an average score of 5 (extremely relevant). The ability to appraise evidence and disseminate outcomes ranged between 4 (very relevant) and 5 (extremely relevant). Figure 3 illustrates nursing faculty perceptions of the relevance of IL skills at the master's level.

**Figure 4:**
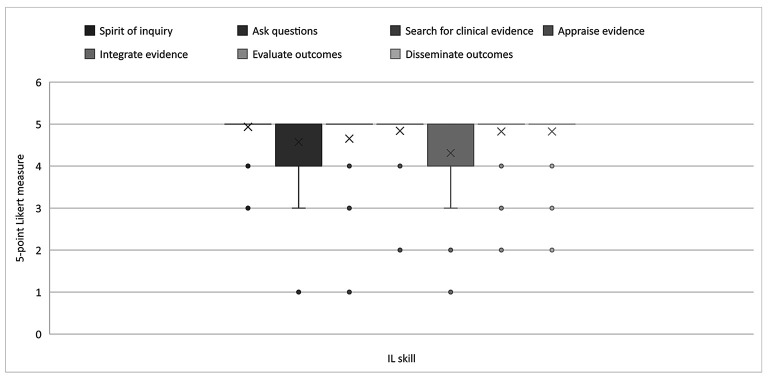
Nursing faculty ratings of relevance of IL skills at the doctoral (PhD) level

The trend continued at the PhD level, where faculty rated the abilities to ask questions and integrate evidence as slightly less relevant than the other 5 skills, with scores averaging between 4 (very relevant) and 5 (extremely relevant). Faculty nearly unanimously agreed that the 5 other skills were extremely relevant, with an average rating of 5. Figure 4 illustrates nursing faculty perceptions of the relevance of IL skills at the PhD level.

**Figure 5:**
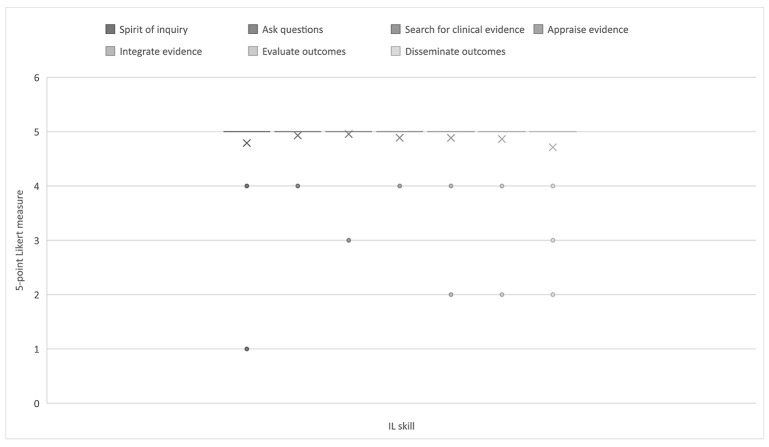
Nursing faculty ratings of the relevance of IL skills at the doctor of nursing (DNP) level

At the DNP level, faculty nearly unanimously agreed that all 7 IL skills were extremely relevant, with all 7 skills receiving an average rating of 5. Figure 5 illustrates nursing faculty perceptions of the relevance of IL skills at the DNP level.

### Nursing faculty integration of IL instruction into course and curriculum design

Most faculty (79%, n=54) indicated that they taught IL principles in their courses. Over half (57%, n=39) stated that they did so because they recognized that students needed it, while 41% (n=28) did so because they felt that IL was part of their skills and interests. Some faculty (32%, n=22) incorporated IL principles because they were an institutional learning outcome, while 21% (n=14) did so for accreditation purposes. Two faculty (3%) listed other reasons, including to utilize problem-based learning and to keep current with evidence-based practice.

While only 12% of faculty (n=8) incorporated a version of the ACRL IL competencies in their courses, they were much more likely to integrate standards from nursing educational associations. Of the 68 faculty respondents, 59% (n=40) included the AACN Essentials, 46% (n=31) included the QSEN competencies, and 9% (n=6) included the TIGER competencies.

Methods for IL instruction included assignments, assigned readings, inclusion of IL competencies in course learning objectives, research sessions with a librarian, modeling of research approaches, and discussion boards.

Some faculty (n=14, 21%) did not include IL principles as part of their instruction. These faculty indicated a variety of reasons for their decision, including that they felt it was not their area of expertise, they expected students to receive IL instruction elsewhere, they did not think to include it, or they left it to librarians to teach.

### Nursing faculty familiarity with ACRL IL standards

Nursing faculty respondents reported little familiarity with any of the ACRL IL competencies. Of the 68 who responded to the question about their awareness of the recently released ACRL Framework for Information Literacy for Higher Education, 79% (n=54) indicated that they were not familiar with the Framework, 9% (n=6) responded that they were familiar with it, and 12% (n=8) were not sure.

Faculty were slightly more familiar with the ACRL Information Literacy Competency Standards for Nursing: 22% (n=15) indicated they were familiar, 62% (n=42) indicated they were not familiar, and 16% (n=11) were not sure.

## DISCUSSION

We predicted nursing faculty would utilize standards from nursing educational associations and the ACRL Information Literacy Competency Standards for Nursing in curriculum development for IL instruction but would be less familiar with the newer, broader Framework for Information Literacy in Higher Education. This hypothesis was partially refuted. While nursing faculty were familiar with nursing educational association standards, they mostly were not familiar with the ACRL Information Literacy Competency Standards for Nursing and were nearly wholly unfamiliar with the Framework.

Nursing faculty were not alone in their lack of familiarity with the Framework. In 2017, Schulte and Knapp surveyed health sciences librarians to determine their familiarity with and use of the Framework [13]. Of the 128 respondents, only 11% had adopted the Framework in their instruction, 35% had future plans to use it, and 54% had no plans to use it, either due to the perceived irrelevance of it to their teaching or their unfamiliarity with it. The results from our survey of faculty, alongside findings from Schulte and Knapp, suggested that a majority of both nursing faculty and nursing librarians were either unfamiliar with the Framework or did not find it useful.

However, examples of the Framework being used to support IL instruction in nursing education have been emerging [14–16]. LeBlanc and Quintiliano described reworking the Conversation, Revision, Authority, and Property (C.R.A.P.) Test, typically used for evaluating sources, to remind students of the Framework [17]. Jacobson and Gibson shared their initial ideas for incorporating the Framework in nursing instruction, including having students participate as information creators via blogs, digital storytelling, and multimedia projects [18]. Young and Hinton provided more than fifty examples of case studies and lesson plans for integrating the Framework into health sciences librarianship [19]. Also, related literature provided ideas that paralleled the Framework without explicitly mentioning it, such as exploring avenues for dissemination [20], using Twitter to stay current and participate in professional conversations [21], and evaluating mobile health apps [22].

Nursing faculty viewed IL instruction as relevant. Despite their unfamiliarity with the Framework, IL competencies were present in the three most used competency models: AACN Essentials, QSEN, and TIGER. Most nursing faculty also reported that they included IL instruction in their individual course curriculums. Our survey results suggested that nursing faculty mostly believed that IL skills were relevant at every educational level. At the associate level, faculty emphasized the relevance of lower-level cognitive skills, such as cultivating a spirit of inquiry, asking burning clinical questions, and searching for the best and most relevant clinical questions. As students continued to the baccalaureate level and higher, they were increasingly expected to execute higher-order cognitive skills, such as the ability to appraise evidence and to disseminate outcomes.

Librarians who are seeking outreach opportunities might use these findings to establish faculty-librarian collaborations [23–26], to work with curriculum committees to weave IL concepts throughout the curriculum, and to scaffold IL instruction so that students continually practice and build on what they know [27, 28]. Our findings emphasized the potential to work with nursing faculty on curriculum development, as a majority of faculty (86%) were at some point involved in curriculum design, but most nursing programs (51%) lacked IL goals.

Miller and colleagues have pointed out that increasing nursing students' IL skills requires understanding each other's discipline-specific content and negotiating content that incorporates both information and nursing practice expertise [25]. By updating the older nursing standards into a framework-style document that acknowledges existing standards of nursing educational associations, we will create a resource that nursing librarians can use to collaborate with nurse educators and integrate IL instructions throughout nursing curriculums at each educational level. We now know that nursing faculty perceptions of the relevance of IL skills change with education levels, and we are particularly interested in developing a resource that supports librarians in communicating the importance of scaffolding IL skills throughout course and program curriculums. We believe that librarian collaboration with faculty who influence their programs' curriculum development offers a scalable and impactful means to integrate IL instruction throughout curriculums to enhance support for one-time librarian visits to classes and librarian co-instruction of courses.

### Future directions

Most nursing faculty are not aware of the Framework but are intentional in using the AACN Essentials and other nursing standards to teach IL. A usable Framework for Information Literacy in Higher Education for Nursing must align with these commonly used nursing standards. More work needs to be done to promote the Framework to health sciences librarians, and the Nursing Information Literacy Framework Working Group will need to be deliberate in marketing and outreach efforts regarding the Framework for Information Literacy in Higher Education for Nursing, both at the draft and adopted stages. Our future marketing efforts will include presentations at both nursing education and librarian conferences and publications in nursing education and librarian journals and newsletters. The AACN Essentials are currently in the process of being revised and a draft version was released in May 2020, which we are using as an opportunity to offer librarian input.

We are also surveying and conducting in-person interviews with stakeholders—such as health sciences librarians, students, and nursing education administrators—to gain broader community insight into how the Framework might be more robustly used. Findings from the literature review, survey, and updated AACN Essentials and feedback from stakeholder interviews will be used to draft the forthcoming Framework for Information Literacy in Higher Education for Nursing.

Our research reveals a gap in literature related to nursing faculty competence and confidence in teaching IL skills. Specifically, how do the existing standards from nursing educational associations influence nurse educators' preparedness to teach IL? And relatedly, how do the existing nursing standards influence students' preparedness to execute IL competencies? Future directions or suggestions for additional research include surveying nursing faculty on their competence or confidence in teaching IL, new nursing faculty about their feelings of preparedness to teach IL, and nursing students to understand how they develop IL competencies during their education.

### Limitations and methodological reflections

Selection bias might have been present in the study, because the results only represented nursing faculty at eight institutions in the United States and might not be applicable on a global scale. Though we aimed to include a diverse demographic by including institutions from different US regions as well as different types of institutions of higher learning—including large research universities, teaching colleges, and community colleges—our results might not be applicable on a national scale. Also, we noted a response bias in that only one respondent currently taught at the associate level.

Respondents were self-selected. Nursing faculty with no interest in IL might have declined to respond, whereas faculty who were already familiar with IL or had a close relationship to their nursing liaison librarian might have been more likely to complete the survey. We attempted to address these limitations by using survey recruitment messaging and in-person recruitment efforts to emphasize how our findings would be used to benefit the nursing education community as a whole.

## CONCLUSION

IL instruction in nursing is taking place even though knowledge of standards and frameworks specific to IL is limited amongst nursing faculty. Integration of IL instruction in nursing programs is mostly achieved through the use of subject-specific nursing education standards such as the Essentials, QSEN competencies, and TIGER competencies. Nursing faculty perceive IL instruction as relevant, and students are expected to master higher-order cognitive skills as they progress through their education. Librarians' understanding of subject-specific standards, changes to faculty perceptions of the relevance of IL skills with educational levels, and the development of IL frameworks that reflect the language and spirit of subject standards are vital to continued meaningful inclusion of IL in nursing programs. These understandings may also be transferable to librarians in other health sciences disciplines.

## Data Availability

All relevant data are available in Purdue e-Pubs, an open repository hosted by Purdue University and can be accessed at https://docs.lib.purdue.edu/lib_fssup/6/.

## SUPPLEMENTAL FILES

**Appendix A:** Survey to measure how nursing faculty approach information literacy instruction

**Appendix B:** Sample survey recruitment email

**Appendix C:** Summary of survey responses
