# Supplementary material for: Evaluating nursing faculty's approach to information literacy instruction: a multi-institutional study
Source: J Med Libr Assoc. 2020 Jul 1;108(3):378–88. doi: 10.5195/jmla.2020.841 (PMC7441900; doi:10.5195/jmla.2020.841)
Supplement: Supplementary file 1 — Appendix A: Survey to measure how nursing faculty approach information literacy instruction [file jmla-108-3-378-s01.pdf]

Journal of the Medical Library Association
Supplemental content to
J Med Libr Assoc. Jul;108(3):dx.doi.org/10.5195/jmla.2020.841
www.jmla.mlanet.org
© McGowan, Cantwell, Conklin, Raszewski, Planchon Wolf, Slebodnik, McCarthy, Johnson 2020

Evaluating nursing faculty's approach to information literacy instruction: a multi-institutional study

Bethany S. McGowan; Laureen P. Cantwell; Jamie L. Conklin; Rebecca Raszewski, AHIP; Julie Planchon Wolf; Maribeth Slebodnik; Sandra McCarthy; Shannon Johnson

APPENDIX A

Survey to measure how nursing faculty approach information literacy instruction

Q1. Introduction: What is information literacy? Information literacy is a set of integrated abilities, encompassing the reflective discovery of information, the understanding of how information is produced and valued, and the use of information in creating new knowledge and participating ethically in communities of learning and practice. These skills are best learned and retained when taught as part of the curriculum and in conjunction with the associated skills of critical thinking, reading, writing, and production. They are developmental and must be introduced, applied, reinforced, and extended throughout a nurse's education and career. The Association of College & Research Libraries (ACRL) Framework for Information Literacy for Higher Education offers educators a structure around which instructional sessions, assignments, courses, and even curricula may be designed. The Framework is organized into six frames that articulate the concepts, knowledge practices, and dispositions central to information literacy: 1. Authority Is Constructed and Contextual, 2. Information Creation as Process, 3. Information Has Value, 4. Research as Inquiry, 5. Scholarship as Conversation, and 6. Searching as Strategic Exploration.

In 2000, a team of librarians established the Standards for Information Literacy in Higher Education, and, in 2013, a discipline-specific version was established: the Information Literacy Competency Standards for Nursing. Recently, ACRL revised the standards to create the Framework for Information Literacy in Higher Education, and, as a result, a new team of librarian-researchers are engaged in revising the nursing standards to create the Framework for Information Literacy in Higher Education for Nursing. This survey is to inform that research.

Q2. Do you consent to being surveyed for this research project? By selecting Yes, you consent that you are willing to answer the questions in this survey.

- o Yes (1)
- o No (2)

Q3. What is the highest level of education that you have obtained?

- o Registered nurse (RN) (1)
- o Bachelor of science in nursing (BSN) (2)
- o Master's of science in nursing (MSN) (3)
- o Master's in another field: (4) ____
- o Doctor of nursing (DNP) (5)
- o Doctorate (PhD) in nursing (6)
- o PhD in another field: (7) ____

SC
Supplemental content to
J Med Libr Assoc. Jul;108(3):dx.doi.org/10.5195/jmla.2020.841
www.jmla.mlanet.org
© McGowan, Cantwell, Conklin, Raszewski, Planchon Wolf, Slebodnik, McCarthy, Johnson 2020

Q4. What body generates the student learning objectives/course outcomes for nursing courses at your institution?

- o I can set my own (1)
- o A curriculum committee in my department/school/college (2)
- o A curriculum committee at the institutional level (3)
- o Program director (4)
- o Not sure (5)
- o Other: (6) ____

Q5. What level of involvement have you had with your program's curriculum? (Select all that apply)

- o Curriculum committee leadership role (1)
- o Currently on curriculum committee (2)
- o Past curriculum committee member (3)
- o Assessment committee position (past or current) (4)
- o Institution-wide/university-wide curriculum committee position (past or current) (5)
- o Accreditation committee (past or current) (6)
- o Other: (7) ____
- o Not involved in curriculum development within my program/school/college/institution (8)

[Logic display:

If What level of involvement have you had with your program's curriculum? (Select all that apply)=Currently on curriculum committee

Or What level of involvement have you had with your program's curriculum? (Select all that apply)=Past curriculum committee member

Or What level of involvement have you had with your program's curriculum? (Select all that apply)=Curriculum committee leadership role]

Q6. Regarding your experience on a curriculum committee, may we contact you again with additional questions we may have?

- o If yes, please email your librarian after completing the survey (1)
- o No, but thanks for asking (2)

Q7. For your nursing program, is there a program goal or outcome related to information literacy (as described in the definition below, if not using that exact term)?

"Information literacy is a set of integrated abilities, encompassing the reflective discovery of information, the understanding of how information is produced and valued, and the use of information in creating new knowledge and participating ethically in communities of learning and practice."

Supplemental content to
J Med Libr Assoc. Jul;108(3):dx.doi.org/10.5195/jmla.2020.841
www.jmla.mlanet.org
© McGowan, Cantwell, Conklin, Raszewski, Planchon Wolf, Slebodnik, McCarthy, Johnson 2020

You or your institution may use phrases like "principles of research" OR "research cycle" to discuss and teach similar concepts.

- o Yes (1)
- o No (2)
- o Not sure (3)

Q8. In what program levels do you teach currently/this academic year/in your current position?

- o Associate level (1)
- o Baccalaureate level (2)
- o Master's level (3)
- o DNP program level (4)
- o PhD in nursing program level (5)

Q9. In what program levels have you taught previously?

- o Associate level (1)
- o Baccalaureate level (2)
- o Master's level (3)
- o DNP program level (4)
- o PhD in nursing program level (5)

Q10. Based on the description below, in your courses, do you teach "information literacy?"

"Information literacy is a set of integrated abilities, encompassing the reflective discovery of information, the understanding of how information is produced and valued, and the use of information in creating new knowledge and participating ethically in communities of learning and practice."

You or your institution may use phrases like "principles of research" OR "research cycle" to discuss and teach similar concepts.

- o Yes (1)
- o No (2)

[Logic display:

If based on the description below, in your courses, do you teach "information literacy?"=Yes]

Q11. If yes, why?

- o Accreditation purposes (1)
- o It is an institutional student learning outcome (SLO) (2)
- o I consider it part of my skillset and/or interests (3)
- o The students need it (4)
- o Other: (5) ____

JMLA Journal of the Medical Library Association
SC
Supplemental content to
J Med Libr Assoc. Jul;108(3):dx.doi.org/10.5195/jmla.2020.841
www.jmla.mlanet.org
© McGowan, Cantwell, Conklin, Raszewski, Planchon Wolf, Slebodnik, McCarthy, Johnson 2020

[Logic display:

If based on the description below, in your courses, do you teach “information literacy?”=No]

Q12. If no, why not?

- Not my area of expertise (1)
- I leave this to librarians (2)
- Barriers (3)
- I expect students receive it elsewhere/prior to my class (4)
- Other: (5) ____

Q13. Are you familiar with the Information Literacy Competency Standards for Higher Education (“the Standards”) or the Information Literacy Standards for Nursing?

- Yes (1)
- No (2)
- Not sure (3)

Q14. Are you aware of the recently released Framework for Information Literacy for Higher Education (“the Framework”)?

- Yes (1)
- No (2)
- Not sure (3)

Q15. Have you incorporated any of the following standards into your teaching practice?

- I have incorporated the Framework (1)
- I have incorporated the Standards (2)
- I have incorporated the Information Literacy Standards for Nursing (3)
- I have incorporated the American Association of Colleges of Nursing (AACN) Essentials (4)
- I have incorporated the Quality and Safety Education for Nurses (QSEN) Competencies (5)
- I have incorporated the Technology Informatics Guiding Education Reform (TIGER) (8)
- Other: (6) ____
- No (7)

Q16. If yes, how do you incorporate information literacy principles or principles of research into your teaching practice?

- Textbook/other readings (1)
- Assignments (2)
- Research session/assistance from librarian (3)
- Discussion boards (4)
- Course learning objectives (5)
- Modeling research approaches for students (6)
- Other: (7) ____

JMLA Journal of the Medical Library Association

SC

Supplemental content to
J Med Libr Assoc. Jul;108(3):dx.doi.org/10.5195/jmla.2020.841
www.jmla.mlanet.org
© McGowan, Cantwell, Conklin, Raszewski, Planchon Wolf, Slebodnik, McCarthy, Johnson 2020

Q17. Using a 1–5 scale (with 5 as the highest score), how relevant are the following information literacy skills or principles of research in each level of nursing education?

	Associate level (1)	Baccalaureate level (2)	Master's level (3)	DNP program level (4)	PhD in nursing program level (5)
Cultivate a spirit of inquiry (1)					
Ask burning clinical questions (2)					
Search for the best and most relevant clinical evidence (3)					
Critically appraise evidence for validity, reliability, and applicability (4)					
Integrate evidence with clinical expertise and patient preferences to implement the best clinical decision (5)					
Evaluate outcomes (6)					
Disseminate outcomes (7)					

Q18. We are working to create a Framework for Information Literacy in Higher Education for Nursing. Would you be interested in reviewing a draft of that document?

- o Yes (1)
- o No (2)

Q19. If yes, please fill out the form at the following: link: xxxx.edu.
