# Supplementary material for: Evaluating nursing faculty's approach to information literacy instruction: a multi-institutional study
Source: J Med Libr Assoc. 2020 Jul 1;108(3):378–88. doi: 10.5195/jmla.2020.841 (PMC7441900; doi:10.5195/jmla.2020.841)
Supplement: Supplementary file 2 — Appendix B: Sample survey recruitment email [file jmla-108-3-378-s02.pdf]

Journal of the Medical Library Association

Supplemental content to
J Med Libr Assoc. Jul;108(3):dx.doi.org/10.5195/jmla.2020.841
www.jmla.mlanet.org
© McGowan, Cantwell, Conklin, Raszewski, Planchon Wolf, Slebodnik, McCarthy, Johnson 2020

Evaluating nursing faculty's approach to information literacy instruction: a multi-institutional study

Bethany S. McGowan; Laureen P. Cantwell; Jamie L. Conklin; Rebecca Raszewski, AHIP; Julie Planchon Wolf; Maribeth Slebodnik; Sandra McCarthy; Shannon Johnson

APPENDIX B

Sample survey recruitment email

Project Title: Tailoring the Framework for Information Literacy for Higher Education to Nursing Education

Hello! We invite you to participate in an online survey that will take approximately ten minutes of your time.

The purpose of this research project is to learn about how information literacy and/or research principles are taught in undergraduate and graduate nursing courses.

There are no direct benefits to you for your participation in this survey. However, the discipline of nursing education and library services supporting nursing education may benefit from the results.

Your participation is entirely voluntary, but please try to answer each question completely. You may withdraw your participation at any time. No personally identifying information is being collected, and your responses to the questions will be anonymous. No one will know whether or not you took this survey.

Please contact the principal investigator, Project Working Group Member (SampleGroupMember@university.edu, 111-111-1111), with any questions about this study.

The plan for this study has been reviewed for its adherence to ethical guidelines by the Purdue University Human Subjects Protection Program/Institutional Review Board. For questions regarding participant rights and ethical conduct of research, contact the vice president of the Purdue University Human Subjects Protection Program/IRB (irb@university.edu, 111-111-1111).

To read the full project details and complete the online survey, please visit www.qualtrics.com.

Thank you for your consideration.

Sincerely,

Working Group Member
