# Supplementary material for: Evaluating nursing faculty's approach to information literacy instruction: a multi-institutional study
Source: J Med Libr Assoc. 2020 Jul 1;108(3):378–88. doi: 10.5195/jmla.2020.841 (PMC7441900; doi:10.5195/jmla.2020.841)
Supplement: Supplementary file 3 — Appendix C: Summary of survey responses [file jmla-108-3-378-s03.pdf]

JMLA Journal of the Medical Library Association

SC
Supplemental content to
J Med Libr Assoc. Jul;108(3):dx.doi.org/10.5195/jmla.2020.841
www.jmla.mlanet.org
© McGowan, Cantwell, Conklin, Raszewski, Planchon Wolf, Slebodnik, McCarthy, Johnson 2020

Evaluating nursing faculty's approach to information literacy instruction: a multi-institutional study

Bethany S. McGowan; Laureen P. Cantwell; Jamie L. Conklin; Rebecca Raszewski, AHIP; Julie Planchon Wolf; Maribeth Slebodnik; Sandra McCarthy; Shannon Johnson

APPENDIX C
Summary of survey responses

Participant demographics	n
Degrees obtained	68
Doctorate (PhD) in nursing	29
PhD in another field	14
Doctorate of nursing (DNP)	10
Master's degree in nursing (MSN)	15
Nursing program levels currently teaching	68*
PhD	8
DNP	25
MSN	23
Bachelor's degree in nursing (BSN)	38
Associate's	1
Not teaching	2
Involvement in program's curriculum	68*
Involved	47
Curriculum committee leadership role	10
Current curriculum committee member	18
Past curriculum committee member	18
Assessment committee member (past or current)	8
Institution-/university-wide curriculum committee member (past or current)	1
Accreditation committee member (past or current)	9
Other	10
No involvement	21

* Faculty could select more than one option. Some faculty are currently teaching in more than one program level and serving in multiple capacities in their program's curriculum.

SC
Supplemental content to
J Med Libr Assoc. Jul;108(3):dx.doi.org/10.5195/jmla.2020.841
www.jmla.mlanet.org
© McGowan, Cantwell, Conklin, Raszewski, Planchon Wolf, Slebodnik, McCarthy, Johnson 2020

Information literacy instruction characteristics	n
Incorporation of instruction on information literacy	68
Yes	54
No	14
Methods for incorporating information literacy or research principles in teaching	54*
Textbook/other readings	34
Assignments	46
Research session/assistance from librarian	25
Discussion boards	17
Course learning objectives	29
Modeling research approaches for students	22
Other	3
Familiarity with the standards or the Information Literacy Standards for Nursing	68
Yes	15
No	42
Not sure	11
Awareness of the Framework	68
Yes	6
No	54
Not sure	8
Inclusion of standards in teaching practices	68*
Includes standards	47
Framework	1
Standards	5
Information Literacy Standards for Nursing	6
American Association of Colleges of Nursing (AACN) Essentials	40
Quality and Safety Education for Nurses (QSEN) Competencies	31
Technology Informatics Guiding Education Reform (TIGER)	6
Other	2
Does not include standards	21

* Faculty could select more than one option. Some faculty are incorporating multiple IL methods and standards into their teaching practices.
